# Understanding Healthcare Workers’ COVID-19 Vaccination Decision-Making as a Dynamic Process: A Qualitative Meta-Synthesis

**DOI:** 10.3390/vaccines14060487

**Published:** 2026-05-30

**Authors:** Hye-Young Jang, Young Ko, Song Yi Han

**Affiliations:** 1 College of Nursing, Hanyang University, Seoul 04763, Republic of Korea; white0108@hanyang.ac.kr; 2 College of Nursing, Gachon University, Incheon 21936, Republic of Korea; moodory@gmail.com; 3 Department of Nursing Science, Sunmoon University, Asan-si 31460, Republic of Korea

**Keywords:** vaccination, healthcare workers, decision-making, meta-synthesis, COVID-19

## Abstract

**Background/Objectives**: Healthcare workers play a critical role in vaccination programs, yet vaccine hesitancy has been widely reported even among this group during the Coronavirus disease 2019 (COVID-19) pandemic. Previous studies have primarily focused on identifying factors associated with vaccine acceptance, offering limited insight into the processes underlying decision-making. This study aimed to synthesize qualitative studies on healthcare workers’ COVID-19 vaccination experiences to develop a comprehensive understanding of their decision-making processes. **Methods**: A qualitative meta-synthesis was conducted using the thematic synthesis approach proposed by Thomas and Harden. Electronic databases including PubMed, Embase, and CINAHL were searched for qualitative studies published up to February 2026. Thirteen studies were included following PRISMA guidelines. Data were analyzed through line-by-line coding, followed by the development of descriptive and analytical themes. **Results**: Four analytical themes were identified: (1) vaccination as a dynamic risk–benefit negotiation process, (2) trust as a central mechanism shaping information interpretation, (3) socially embedded and relationally negotiated decision-making, and (4) moral identity as a driver of vaccination behavior. Healthcare workers’ vaccination decision-making was not a static choice but an evolving process shaped by continuous appraisal of risks and benefits, filtered through trust in information and institutions, influenced by social interactions, and guided by professional identity and ethical responsibility. **Conclusions**: Healthcare workers’ vaccination decision-making is a multidimensional process embedded in cognitive, social, and ethical contexts. Interventions should move beyond individual-level approaches and instead focus on building trust, leveraging social networks, and reinforcing professional identity with implications for future public health crises.

## 1. Introduction

Coronavirus disease 2019 (COVID-19) represents one of the most extensive and prolonged global health crises in modern history, resulting in substantial transformations across healthcare systems and societies. During the pandemic, vaccination emerged as a central public health strategy, alongside non-pharmaceutical interventions, to control the spread of infection [1,2].

The rapid development and authorization of COVID-19 vaccines marked an unprecedented departure from traditional vaccine development timelines, which typically involve prolonged clinical validation processes [3]. While this accelerated process enabled timely pandemic response, it also generated uncertainty regarding vaccine safety, potential side effects, and long-term outcomes. These concerns were particularly salient among healthcare workers, who were required to make complex decisions balancing scientific evidence with personal risk perception [4].

Vaccination is not merely a preventive health behavior but a multifaceted decision-making process shaped by risk perception, trust, value judgments, and social and ethical responsibility [5]. The World Health Organization identified vaccine hesitancy as one of the top global health threats even prior to the COVID-19 pandemic [6]. Healthcare workers occupy a unique position in this context, simultaneously acting as vaccine recipients, recommenders to patients and the public, and implementers of health policies. Consequently, their vaccination decisions are situated within complex professional, ethical, and social contexts that differ from those of the general population [7].

Previous research on healthcare workers’ vaccination has predominantly employed quantitative approaches, focusing on factors such as intention, attitudes, knowledge, beliefs, and sociodemographic characteristics [8,9,10]. While these studies have contributed to identifying determinants of vaccine acceptance, they offer limited insight into how healthcare workers actually experience, interpret, and negotiate their decisions in real-world contexts. In particular, they fail to capture the dynamic processes through which hesitation, conflict, justification, and meaning-making unfold over time.

Qualitative studies have provided valuable insights into healthcare workers’ lived experiences of vaccination decision-making, revealing the contextual and interpretive nature of these processes. However, these studies are often limited to specific countries, healthcare systems, or time points, resulting in fragmented and context-bound findings. As such, there remains a lack of integrative understanding of how vaccination decision-making is constructed across diverse settings.

Qualitative meta-synthesis offers a methodological approach to move beyond individual study findings by identifying patterns, similarities, and variations across studies, thereby generating higher-order conceptual insights [11,12,13]. In particular, it enables the development of a more comprehensive and theoretically informed understanding of complex phenomena such as vaccination decision-making.

Although the acute phase of the COVID-19 pandemic has subsided, ongoing challenges such as emerging infectious diseases, booster vaccination programs, and healthcare worker immunization policies remain highly relevant. In this context, a systematic and integrative understanding of healthcare workers’ vaccination decision-making is essential not only for interpreting past experiences but also for informing future public health strategies.

Therefore, this study aims to synthesize qualitative evidence on healthcare workers’ COVID-19 vaccination experiences to identify shared patterns and underlying meaning structures in their decision-making processes. This can be utilized as evidence for establishing vaccination policies for healthcare professionals, developing educational programs, and formulating nursing intervention strategies in the future.

## 2. Materials and Methods

### 2.1. Research Design

This study is a qualitative meta-synthesis that integrates and interprets findings from qualitative studies exploring healthcare workers’ vaccination decision-making experiences.

### 2.2. Literature Search

The literature search and study selection were conducted in accordance with the Preferred Reporting Items for Systematic Reviews and Meta-Analyses (PRISMA) guidelines. Studies published up to February 2026 were included.

Electronic databases including PubMed, Embase, and CINAHL were searched. The search strategy combined the following keywords: “healthcare personnel” AND “COVID-19” AND “vaccination decision” AND “qualitative research.” No restrictions were placed on publication year.

### 2.3. Eligibility Criteria and Study Selection

The inclusion criteria were as follows: (1) studies exploring vaccination decision-making experiences, (2) studies involving healthcare workers, (3) studies employing qualitative research methods, and (4) studies published in English.

The exclusion criteria were: (1) studies focusing on vaccines other than COVID-19 (e.g., influenza or HPV vaccines), because COVID-19 vaccination occurred within a unique pandemic context characterized by accelerated vaccine development, emergency authorization, and heightened social and political uncertainty, (2) studies including participants other than healthcare workers (e.g., patients or family members), (3) studies addressing attitudes, knowledge, perceptions, or acceptance levels without exploring the process through which healthcare workers interpreted, negotiated, justified, or constructed vaccination decisions, (4) studies using quantitative methods, and (5) non-original articles.

**Figure 1:**
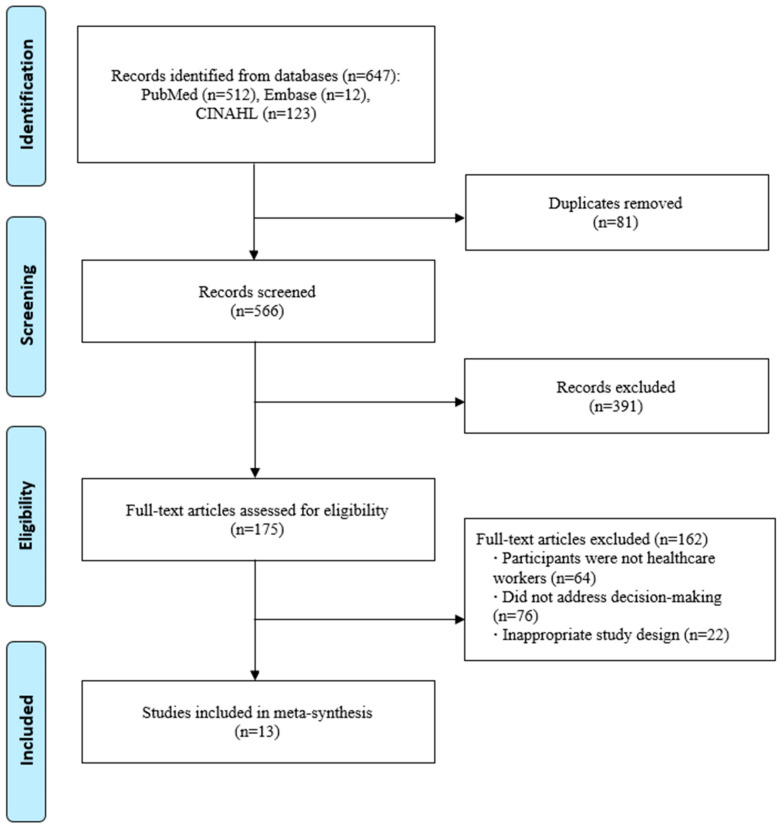
PRISMA flow diagram of the study selection process.

A total of 647 studies were identified (PubMed: 512, CINAHL: 123, Embase: 12). After removing 81 duplicate records identified primarily through overlap among the PubMed, CINAHL, and Embase databases, 566 studies were screened based on titles and abstracts. Of these, 391 studies were excluded. The full texts of 175 studies were assessed for eligibility, and 162 studies were excluded. Finally, 13 studies were included in the synthesis. The PRISMA flow diagram illustrating the study selection process is shown in Figure 1.

### 2.4. Quality Appraisal

The methodological quality of the included studies was assessed using the Critical Appraisal Skills Programme (CASP) [14] by two independent reviewers. The CASP checklist consists of ten questions evaluating the rigor, credibility, and relevance of qualitative research.

The two reviewers independently assessed each study and compared their evaluations. Minor discrepancies in several appraisal items were resolved through iterative discussion until consensus was achieved. All 13 studies were judged to have acceptable methodological rigor (Table 1).

### 2.5. Data Analysis and Synthesis

The aim of qualitative meta-synthesis is to achieve a deeper understanding of a research phenomenon by integrating findings from individual studies [11,12,13]. Rather than merely summarizing or categorizing findings, qualitative synthesis involves an interpretive process that identifies patterns, commonalities, and variations to generate higher-order concepts that may not be evident in individual studies alone [12,28]. Data analysis was supported using MAXQDA 2020 software (VERBI Software, Berlin, Germany) to facilitate coding and data management.

This study employed the three-stage thematic synthesis approach proposed by Thomas and Harden [29]. In the first stage, the findings of the included studies were read repeatedly, and line-by-line coding was conducted to generate initial codes grounded in the original data. This process focused on capturing meanings and interpretations presented in the results sections of the primary studies.

In the second stage, the initial codes were compared and grouped based on similarities and differences. Through this process, descriptive themes were developed to represent patterns in the data while remaining close to the original findings.

In the third stage, analytical themes were generated through an interpretive process that moved beyond the descriptive level to provide higher-order explanations of healthcare workers’ vaccination decision-making. This stage involved synthesizing relationships between descriptive themes and integrating variations to construct more abstract conceptual understandings.

Throughout the analysis, multiple researchers independently reviewed the data and engaged in iterative discussions to refine codes and themes until consensus was reached. Any discrepancies in interpretation were resolved through discussion, thereby enhancing the credibility and methodological rigor of the synthesis.

### 2.6. Ethical Consideration

This study was exempted from review by the Institutional Review Board (IRB) because it involved the synthesis of previously published studies (IRB No. SM-202601-001-1).

## 3. Results

### 3.1. Overview of Included Studies and Synthesis Approach

The characteristics of the 13 studies included in this synthesis are presented in Table 2. Five studies were conducted in Asian countries, seven in European countries, and one in an African country. A total of 362 participants were included across the studies.

The included studies used varying terms such as healthcare workers, healthcare professionals, and healthcare providers. In this review, we retained the terminology used in the original studies to preserve conceptual consistency. Although these terms may differ in scope, participants across studies primarily consisted of frontline clinical personnel involved in direct patient care. Therefore, the variation in terminology was considered unlikely to substantially influence the synthesis findings.

Ten studies employed thematic analysis, while the remaining studies used other qualitative analytical approaches such as content analysis and grounded theory. All studies used interview-based qualitative data collection methods, including individual interviews and focus groups.

### 3.2. Main Findings

The analytical themes were developed through an iterative process of grouping descriptive themes and underlying codes. These analytical themes represent an interpretive synthesis that moves beyond the descriptive level to provide a higher-order understanding of healthcare workers’ vaccination decision-making. An example of how codes were translated into descriptive and analytical themes is presented in Table 3.

#### 3.2.1. Development of Descriptive Themes

Through line-by-line coding of the findings from the included studies, eight descriptive themes were generated. These themes represented patterns in healthcare workers’ experiences and perceptions regarding COVID-19 vaccination and were closely grounded in the original data.

The descriptive themes included: (1) perceived risk and safety concerns, (2) uncertainty and evolving knowledge, (3) trust and distrust in information sources, (4) influence of social interactions, (5) workplace and organizational context, (6) professional responsibility and role expectations, (7) personal health beliefs and perceived immunity, and (8) moral and ethical considerations. These descriptive themes served as the basis for the development of higher-order analytical themes through an interpretive process.

#### 3.2.2. Analytical Themes

The analytical themes were developed by interpreting and integrating the descriptive themes. While descriptive themes reflected patterns in the data, the analytical themes represent higher-order interpretations that explain how healthcare workers construct and negotiate their vaccination decisions.

1. Vaccination as a Dynamic Risk–Benefit Negotiation Process

This theme was developed from descriptive themes related to perceived risk, safety concerns, uncertainty, and personal health beliefs. Healthcare workers’ vaccination decisions were understood not as fixed judgments but as a dynamic process of continuously comparing and re-evaluating perceived risks and expected benefits. This pattern was consistently reported across multiple studies [15,16,17,18,19].

Participants weighed concerns about potential side effects, uncertainty regarding long-term safety, and doubts about vaccine effectiveness against the perceived benefits of protecting themselves, their families, and their patients [20,21,22]. This appraisal evolved over time; in the early stages, participants tended to adopt a cautious stance, actively seeking information or observing others’ experiences [15,23,24]. As new information and social experiences accumulated, initial judgments were revisited. For some, this process led to vaccine acceptance, whereas for others it resulted in continued hesitation or sustained skepticism [16,17,25,26].

Tensions between individual autonomy and external pressures also played a significant role in shaping decision-making. In some cases, organizational recommendations or policies facilitated vaccine uptake [20,22], while in others such pressures were perceived as infringements on autonomy, leading to resistance [19,21,27].

2. Trust as a Central Mechanism Shaping Information Interpretation

This theme emerged from descriptive themes concerning trust in information sources, conflicting information, and prior experiences with healthcare systems. Although healthcare workers were exposed to a wide range of information, they did not accept it at face value; rather, they interpreted information through a lens of trust in its source. This pattern was consistently observed across studies [15,16,22,23,24]. The same information could be interpreted differently depending on the level of trust. When trust was high, information served as reassurance, whereas low trust amplified uncertainty and suspicion [17,19,20].

The information environment was complex, characterized by the coexistence of formal guidelines and informal sources. Information was disseminated through social media, peer networks, and personal experiences, and conflicting messages as well as shared negative experiences contributed to confusion [16,18,25,26]. In particular, inconsistencies across institutional sources made it difficult for healthcare workers to assess the credibility of information [21,24].

Information interpretation was also closely linked to individuals’ social and historical experiences. In some studies, prior experiences with healthcare systems or perceptions of social inequality led to skepticism toward institutions and a more critical interpretation of official recommendations [19,22,27].

3. Socially Embedded and Relationally Negotiated Decision-Making

This theme was derived from descriptive themes related to social interactions, peer influence, and workplace context. Vaccination decision-making was not an isolated individual process but was embedded within social relationships and interactions. This pattern was consistently reported across studies [15,18,21,24,26].

Healthcare workers adjusted their decisions through conversations with colleagues, observation of others’ behaviors in the workplace, and input from family members [27,30,31]. In particular, colleagues’ vaccination behaviors functioned as key reference points, and others’ experiences directly influenced individual decision-making [16,22,25].

The social environment exerted both facilitating and inhibiting effects. Positive vaccination experiences promoted acceptance, whereas reports of adverse effects or skeptical opinions reinforced hesitation [19,21,27]. This reflects how both information and emotions spread within social networks. Informal interactions and the collective atmosphere within the workplace played a particularly important role. In some cases, conversations among colleagues and shared experiences exerted a stronger influence than formal policies or guidelines [17,18,24], highlighting that vaccination decisions are constructed within collective contexts rather than solely at the individual level.

4. Moral Identity as a Driver of Vaccination Behavior

This theme was constructed from descriptive themes reflecting professional responsibility, ethical obligations, and moral reasoning. Across studies, healthcare workers perceived vaccination not merely as a personal choice but as an issue closely tied to professional identity and moral responsibility, which significantly influenced their decision-making [15,16,19,22,26]. Healthcare workers emphasized their duty to protect patients, particularly vulnerable populations, and understood vaccination as part of their professional role [20,21,23]. This sense of responsibility often shifted decision-making toward prioritizing collective protection over individual concerns.

In several studies, healthcare workers who initially hesitated ultimately accepted vaccination through a process of re-evaluating their professional roles and ethical responsibilities [17,24,25]. This indicates that decision-making involves not only risk assessment but also identity-based reconstruction. Furthermore, healthcare workers recognized that their decisions could affect not only patients but also colleagues and the broader community. Vaccination was thus understood as an act of fulfilling social responsibility [18,27], contributing to the redefinition of vaccination from an individual choice to a component of professional role performance.

## 4. Discussion

This meta-synthesis contributes to a comprehensive understanding of healthcare workers’ COVID-19 vaccination decision-making as a complex and dynamic process. The findings suggest that vaccination decisions are not fixed but evolve through continuous risk–benefit appraisal, shaped by trust in information and institutions, embedded within social and relational contexts, and influenced by professional identity and moral responsibility.

Healthcare workers’ vaccination decision-making emerged as a dynamic negotiation process rather than a one-time act of acceptance or refusal. In this study, healthcare workers continuously weighed concerns about potential side effects against the perceived benefits of protecting patients, resulting in a trajectory that evolved from initial hesitation to eventual acceptance or sustained skepticism. This fluidity supports Larson et al.’s [32] conceptualization of vaccine acceptance as a continuum.

Despite their professional knowledge, healthcare workers expressed substantial concerns regarding vaccine safety, consistent with previous findings [33], that medical expertise does not necessarily guarantee vaccine acceptance. These findings suggest that decision-making is not merely a function of scientific evidence but reflects a psychological negotiation between professional responsibility and personal uncertainty. The tendency to delay decisions while gathering information may be interpreted, in light of self-determination theory [30], as an effort to preserve professional autonomy and develop internally grounded confidence rather than comply with external pressures. As highlighted by Suran [33], transparent communication and open discussion of potential risks are essential to enabling healthcare workers to function as active decision-makers. This implies that, rather than relying on mandates, policies should provide sufficient time and psychological space for individuals to construct meaning and make autonomous decisions. Accordingly, healthcare workers should not be viewed merely as passive recipients of guidelines; instead, their uncertainty should be acknowledged, and their cautious deliberation should be supported through psychologically safe environments that facilitate informed and autonomous decision-making.

Trust has long been recognized as a key determinant of vaccine acceptance. However, the present findings extend this understanding by demonstrating that trust functions not merely as a single factor but as a central interpretive mechanism that mediates the entire process through which healthcare workers evaluate information, construct understanding, and translate it into action. Despite their professional expertise, participants did not accept information at face value; rather, they reinterpreted it through what can be described as a “filter of trust” based on the credibility of the information source. This aligns with Hendriks et al. [34], who suggest that individuals evaluate not only the expertise of information providers but also their benevolence and integrity.

These findings may be understood through the lens of sensemaking theory [31,35], which suggests that individuals do not passively receive information as objective facts but actively construct meaning through social interaction and contextual interpretation, particularly under conditions of uncertainty. During the COVID-19 pandemic, healthcare workers were continuously exposed to rapidly evolving and sometimes conflicting information, requiring them to repeatedly interpret and reinterpret vaccine-related knowledge. Consequently, information was interpreted not only through scientific evidence itself, but also through perceptions of institutional credibility, relational trust, and awareness of collectively shared norms and social meaning systems within groups.

Furthermore, identical information could lead to reassurance or skepticism depending on the level of trust in institutions, indicating that past experiences of systemic inequality or negative interactions with healthcare systems may shape current interpretations. This finding is consistent with Goldenberg [36], who argues that vaccine hesitancy is rooted not in a lack of scientific literacy but in distrust toward institutions. Consequently, scientific evidence alone is insufficient if it fails to overcome the barrier of distrust. Therefore, communication strategies should move beyond one-way dissemination of numerical data and instead prioritize rebuilding trust by conveying transparency, integrity, and benevolence, as emphasized by Hendriks et al. [34]. This further suggests that effective vaccination communication should extend beyond the mere provision of accurate information and support opportunities for collective meaning-making within trusted relational and organizational environments.

Vaccination decision-making was also deeply socially embedded, extending beyond individual cognition into interactions with colleagues and organizational culture. Healthcare workers relied heavily on peers’ vaccination status and experiences as key reference points, and informal conversations in shared spaces such as corridors or break rooms often exerted greater influence than formal guidelines. This finding is consistent with previous research indicating that peer networks influence vaccine acceptance among healthcare workers [37]. It also reflects the principle of social proof [38], whereby individuals rely on others’ behavior as a guide in uncertain situations. These findings suggest that strategies to promote vaccine uptake should leverage peer influence and social networks, such as opinion leaders and trusted colleagues, rather than relying solely on top-down communication approaches [37].

The final driver of decision-making was derived from professional identity and ethical responsibility, extending beyond individual well-being. Healthcare workers who initially hesitated often experienced a turning point as they re-evaluated their professional role and responsibility to protect patients and the broader community, ultimately leading to vaccine acceptance. This transformation can be explained by moral identity theory [39], which posits that individuals are more likely to act in ways that align with values central to their identity. The recognition that refusing vaccination could harm patients or colleagues may generate moral tension, reinforcing professional obligation. Consequently, vaccination can be understood not merely as a health behavior but as an existential act affirming one’s professional identity. Therefore, vaccination policies should move beyond incentives and instead focus on ethical reframing that strengthens professional pride, altruism, and moral responsibility.

This study provides an integrative and in-depth understanding of healthcare workers’ vaccination decision-making by applying a rigorous thematic synthesis to qualitative evidence across diverse contexts. Importantly, vaccination decision-making was conceptualized not as a static outcome determined by isolated factors, but as a dynamic, socially embedded, and interpretive process. In particular, this study highlights the central role of trust as a mechanism shaping interpretation, the relational nature of decision-making, and the influence of professional identity in transforming individual concerns into collective responsibility.

Several limitations should be considered. The included studies were conducted within specific cultural and healthcare contexts, which may limit the transferability of the findings. In addition, all studies included in this synthesis were conducted during or after the COVID-19 pandemic, reflecting vaccination decision-making under unprecedented public health circumstances. Prior to the pandemic, vaccination decision-making was often situated within relatively stable routine immunization contexts, such as seasonal influenza or HPV vaccination programs [5]. These differences suggest that healthcare workers’ vaccination decision-making is influenced not only by individual risk perception but also by broader social and policy contexts [5,36]. In contrast, COVID-19 vaccination occurred under unique conditions characterized by accelerated vaccine development, emergency authorization, rapidly evolving scientific evidence, widespread misinformation, and heightened social and political uncertainty [3,33]. These conditions may have amplified uncertainty, intensified distrust, and increased the importance of relational and ethical dimensions in healthcare workers’ decision-making processes. Therefore, caution is warranted when applying these findings to other vaccination contexts.

Nevertheless, several mechanisms identified in this synthesis—including trust-based interpretation of information, peer influence, and professional ethical responsibility—are unlikely to be unique to COVID-19 vaccination alone. Rather, the pandemic may have magnified underlying decision-making processes that also existed within routine vaccination settings. Accordingly, the findings of this study should be interpreted as reflecting both enduring mechanisms of vaccination decision-making and the unique contextual pressures associated with the COVID-19 pandemic. Finally, variations in study design and reporting quality may have influenced the synthesis process.

Based on these findings, several implications can be suggested. First, interventions should move beyond simply providing information and instead focus on building trust in institutions and communication processes. Second, strategies should leverage peer influence and social networks within healthcare settings to promote positive vaccination behaviors. Third, policies should carefully balance public health goals with respect for individual autonomy to avoid resistance. Finally, strengthening professional identity and ethical responsibility may serve as an effective approach to encourage vaccine acceptance among healthcare workers.

## 5. Conclusions

This study demonstrates that healthcare workers’ vaccination decision-making is a multidimensional process shaped by the complex interplay of cognitive, social, and ethical dimensions. In the early stages, decision-making is primarily driven by individual-level risk–benefit appraisal (cognitive level). However, this appraisal is amplified or attenuated through peer norms and interactions within organizational contexts (social level), and ultimately translated into action through professional identity and a sense of moral responsibility (ethical level). A key finding of this study is the identification of a “filter of trust,” which functions as a critical mediating mechanism in the process through which information is translated into behavior.

These findings suggest that interventions aimed at increasing vaccination uptake should move beyond a narrow focus on individual-level determinants and instead incorporate dynamic and relational dimensions. Furthermore, public health strategies targeting healthcare workers should shift away from a fragmented approach centered on information provision toward a more comprehensive framework that strengthens trust-based social capital within healthcare organizations and fosters moral efficacy grounded in professional identity.

## Figures and Tables

**Table 1:** Quality Assessment.

	Authors [Ref.]	Balay-odao et al. [15]	Heyerdahl et al. [16]	Marså et al. [17]	Ottonello et al. [18]	Siu [19]	Geva et al. [20]	Madran et al. [21]	Ng et al. [22]	Shiferie et al. [23]	Thampy et al. [24]	Tunisi et al. [25]	Woodhead et al. [26]	Yoon et al. [27]
	Items													
1	Was there a clear statement of the aims of the research?	Yes	Yes	Yes	Yes	Yes	Yes	Yes	Yes	Yes	Yes	Yes	Yes	Yes
2	Is a qualitative methodology appropriate?	Yes	Yes	Yes	Yes	Yes	Yes	Yes	Yes	Yes	Yes	Yes	Yes	Yes
3	Was the research design appropriate to address the aims of the research?	Yes	Yes	Yes	Yes	Yes	Yes	Yes	Yes	Yes	Yes	Yes	Yes	Yes
4	Was the recruitment strategy appropriate to the aims of the research?	Yes	Yes	Yes	Yes	Yes	Yes	Yes	Yes	Yes	Yes	Yes	Yes	Yes
5	Was the data collected in a way that addressed the research issue?	Yes	Yes	Yes	Yes	Yes	Yes	Yes	Yes	Yes	Yes	Yes	Yes	Yes
6	Has the relationship between researcher and participants been adequately considered?	Can’t tell	Can’t tell	Can’t tell	No	No	Yes	Can’t tell	Yes	Can’t tell	Can’t tell	Can’t tell	Can’t tell	Can’t tell
7	Have ethical issues been taken into consideration?	Yes	Yes	Yes	Yes	Yes	Yes	Yes	Yes	Yes	Yes	Yes	Yes	Yes
8	Was the data analysis sufficiently rigorous?	Yes	Yes	Yes	Yes	Yes	Yes	Yes	Yes	Yes	Yes	Yes	Yes	Yes
9	Is there a clear statement of findings?	Yes	Yes	Yes	Yes	Yes	Yes	Yes	Yes	Yes	Yes	Yes	Yes	Yes
10	How valuable is the research?	Yes	Yes	Yes	Yes	Yes	Yes	Yes	Yes	Yes	Yes	Yes	Yes	Yes

**Table 2:** Characteristics of Included Studies (*n* = 13).

Author [Ref.]	Country	Aim	Design	Participants (*n*)	Data Collection	Analysis Method
Balay-odao et al. [15]	Philippines	To explore COVID-19 vaccine hesitancy among Filipino nurses	Descriptive phenomenological qualitative study	Nurses (*n* = 13)	In-depth interviews	Thematic analysis
Heyerdahl et al. [16]	Belgium	To explore how divergent vaccine sentiments affected HCWs’ relations and decisions	Qualitative study	HCWs (*n* = 74)	In-depth interviews & Focus groups	Thematic analysis
Marså et al. [17]	Denmark	To explore experiences of vaccine-hesitant healthcare professionals	Qualitative study	HCPs (*n* = 18)	Semi-structured interviews	Thematic analysis
Ottonello et al. [18]	Italy	To explore nurses’ attitudes towards COVID-19 vaccines	Qualitative descriptive study	Nurses (*n* = 30)	Focus groups	Thematic analysis
Siu [19]	Hong Kong	To explore barriers and moral struggle related to COVID-19 vaccination	Qualitative descriptive study	Nurses (*n* = 35)	Semi-structured interviews	Thematic analysis
Geva et al. [20]	Israel	To explore factors shaping healthcare professionals’ COVID-19 vaccine decision-making	Qualitative study	HCPs (*n* = 12)	Semi-structured interviews	Hybrid thematic analysis
Madran et al. [21]	Turkey	To explore reasons for COVID-19 vaccine hesitancy among HCWs	Qualitative study	HCWs (*n* = 23)	Semi-structured interviews	Thematic analysis
Ng et al. [22]	Hong Kong	To explore experiences of COVID-19 vaccination among primary healthcare workers	Qualitative descriptive study	HCWs (*n* = 28)	Semi-structured interviews	Thematic analysis
Shiferie et al. [23]	Ethiopia	To explore reasons for COVID-19 vaccine hesitancy among healthcare providers	Phenomenological study	HCPs (*n* = 20)	In-depth interviews	Content analysis
Thampy et al. [24]	India	To explore phenomenology of COVID-19 vaccine skepticism	Mixed-methods study (qualitative phase)	HCWs (*n* = 30)	In-depth interviews	Grounded theory approach
Tunisi et al. [25]	Italy	To explore COVID-19 vaccination decision-making among HCWs in hematology	Qualitative descriptive study	HCWs (*n* = 21)	In-depth interviews	Content analysis
Woodhead et al. [26]	UK	To explore vaccine decision-making among racial/ethnic minority HCWs	Qualitative study	HCWs (*n* = 25)	Semi-structured interviews	Thematic analysis
Yoon et al. [27]	Singapore	To explore multifactorial influences on COVID-19 vaccination decisions	Qualitative study	HCWs (*n* = 33)	Semi-structured interviews	Thematic analysis

HCWs = healthcare workers; HCPs = healthcare professionals or healthcare providers, as defined in the original studies.

**Table 3:** Development of analytical themes from descriptive themes and codes.

Code	Descriptive Theme	Analytical Theme
perceived low risk perceived susceptibility fear of side effects long-term uncertainty perceived vaccine efficacy benefit perception	Perceived risk and benefit appraisal	Vaccination as a dynamic risk–benefit negotiation process
autonomy freedom of choice resistance to mandate perceived coercion	Autonomy and resistance to coercion	
individual-centered decision community-centered decision perspective shift changing opinion	Decision orientation and transition	
trust in health authority distrust in government pharma distrust distrust in vaccine development historical mistrust structural racism	Trust and distrust in institutions and science	Trust as a central mechanism shaping information interpretation
misinformation exposure social media influence information insufficiency conflicting information amplification of adverse events	Influence of information environment	
peer influence family influence social norms stigma pressure	Social and relational influences	Socially embedded and relationally negotiated decision-making
policy environment access supply issues inequality workplace context	Structural and contextual influences	
duty of care protecting patients role model identity ethical obligation	Professional identity and moral responsibility	Moral identity as a driver of vaccination behavior

## Data Availability

No new data were created or analyzed in this study. Data sharing is not applicable to this article.
